# A Concise Review of Gold Nanoparticles-Based Photo-Responsive Liposomes for Controlled Drug Delivery

**DOI:** 10.1007/s40820-017-0166-0

**Published:** 2017-10-31

**Authors:** Malathi Mathiyazhakan, Christian Wiraja, Chenjie Xu

**Affiliations:** School of Chemical and Biomedical Engineering, Nanyang Technological University, 62 Nanyang Drive, Singapore, 637459 Singapore

**Keywords:** Photo-responsive liposome, Controlled release, Drug delivery, Gold nanoparticles

## Abstract

The focus of drug delivery is shifting toward smart drug carriers that release the cargo in response to a change in the microenvironment due to an internal or external trigger. As the most clinically successful nanosystem, liposomes naturally come under the spotlight of this trend. This review summarizes the latest development about the design and construction of photo-responsive liposomes with gold nanoparticles for the controlled drug release. Alongside, we overview the mechanism involved in this process and the representative applications. 
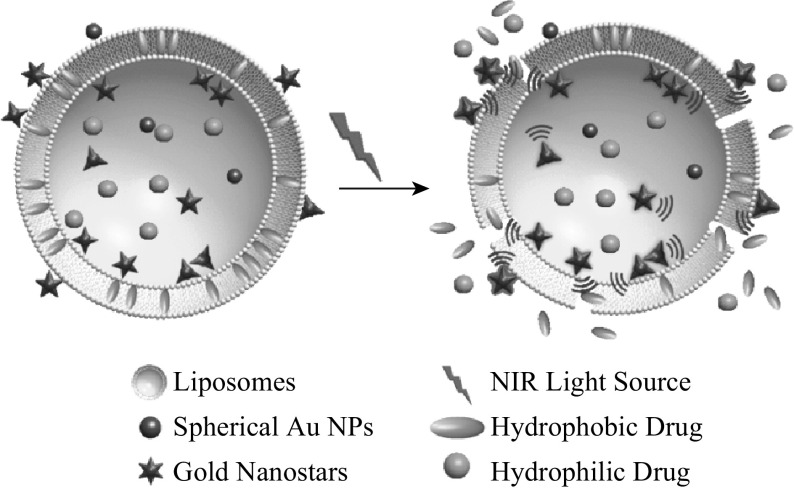

## Highlights

- Photo-responsive liposomes are powerful carriers for topical and transdermal drug delivery to superficial tissues like skin, eyes, and mucous membranes.
- Photo-responsive liposomes can be built with gold nanoparticles, which allow the use of near-infrared lights as the light source for deep tissue penetration and low phototoxicity.
- This review summarizes the latest development and scientific understanding of this complex in delivering drugs for tuning cell signaling pathway and treating cancer.

## Background

Liposomes are man-made vesicles consisting of a phospholipid bilayer [1]. Since the 1970s, liposomes have been extensively investigated as drug carriers because of their biocompatibility and ability to deliver both hydrophilic and hydrophobic therapeutic agents. They are the most successful drug carriers with over 40 liposome-based therapeutics either in market (e.g., Doxil^®^, Ambisome^®^, and DepoDur™) or in various phases of clinical trials [2, 3].

Early generations of liposome-based formulations consist of the inert carriers and the therapeutics. They are delivered orally/systematically and passively accumulate at the disease areas. In cancer therapy, they accumulate at tumor area through enhanced permeation and retention (EPR) effect, which exploits the leaky vasculature and poor lymphatic drainage of solid tumors [4]. However, these conventional liposomes are far from ideal in terms of drug bioavailability. Ideally, the delivery system should deposit a sufficient amount of drugs at the target site at the right time while minimizing the drug concentrations at the non-target tissues.

To meet this need, tremendous efforts have been made to develop drug delivery systems that can release therapeutics in response to the changes in the microenvironment [5–7]. The change of microenvironment can be due to the internal factors like pH and enzymes, or the external factors such as light and temperature. This change is then sensed by the sensing components in the liposomes, which destabilize the liposomes and result the release of liposome-entrapped drugs.

## Synthesis of Gold Nanoparticle-Liposomes

External triggers for destabilizing liposomes include heat, light, and ultrasound. Heat can also be generated using alternating magnetic field. It is noteworthy that thermosensitive liposomes (ThermoDox^®^, currently in Phase 3 clinical trials) are thus far the best-studied example of triggerable nanoparticles [8]. Among different external triggers, light provides unparalleled spatio-temporal control and is easy to access and patient-friendly. Thus, liposomes responsive to light have great potentials in topical and transdermal drug delivery and can readily be used for delivering drugs to superficial tissues like skin, eyes, and mucous membranes.

To respond to the light, photosensitizers have to be incorporated into the liposomes to trigger the release. Although there are a wide range of photosensitizers to choose including photosensitive lipids/surfactants, photosensitive molecular dyes, or photosensitive nanoparticles, the following criteria should be met: (1) the incorporation of photosensitizers does not disturb the physical and chemical properties of the liposomes; (2) under the stimulus, the photosensitizers disrupt the liposomes effectively and efficiently; and (3) the stimulus has minimal damage to the tissue and organ.

This review focuses on the use of gold nanoparticles (Au NPs). Au NPs have their unique optical properties that are tunable by changing the size, shape, surface chemistry, or aggregation state. They are also safe and biocompatible. When being made in suitable size and shape, Au NPs allow the use of near-infrared (NIR) lights as the light source that permits deep tissue penetration and low phototoxicity. The unique optical and electronic properties also allow them to act as the contrast agents of several imaging modalities (e.g., photoacoustic imaging) for revealing the spatio-temporal biodistribution of the liposomes.

**Fig. 1:**
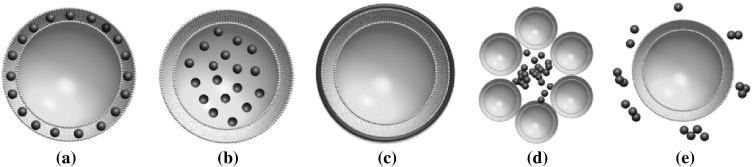
Five types of Au NP-liposome complex: **a** Au NPs are within lipid bilayer of liposomes; **b** Au NPs are in aqueous core of liposomes; **c** Au NPs are on the surface of the liposomes; **d** Au NPs assemble as aggregates with liposomes; and **e** Au NPs are free in liposome solution

Au NPs can be incorporated within the liposomes using methods like reverse evaporation (REV), thin film hydration (TFH), interdigitation-fusion, and lipid vesicle metallization. Most of these methods yield multilamellar vesicles first, which are then extruded to unilamellar vesicles using size extruder. The unencapsulated Au NPs are removed by purification techniques such as gel filtration chromatography, ultra-filtration, ultra-centrifugation, or dialysis [9]. Based on the position of Au NPs, the complexes can be grouped into five subgroups—within the lipid bilayer, in the aqueous core, on the surface of the liposomes, aggregates with liposomes, and free in liposome solution (Fig. 1, Table 1).

**Table 1:** Five kinds of Au NP-liposome complex systems

	Properties of Au NPs	Lipid composition	Encapsulated molecules	Laser	References
Within the lipid bilayer of liposomes	3–4 nm Au NPs, coated with stearylamine	DPPC	Nil	Nil	[10]
	2–3 nm Au NPs coated with hexanethiol	DSPC/DPPC	Calcein	250 nm UV	[11]
	5 nm Au NPs coated with di-2-ethylhexyl sulfosuccinate	Lecithin, cholesterin	Alkaloid berberine	250 nm UV	[12]
	PTX-PEG400 coated Au NPs	Soya phosphatidylcholine, cholesterol	PTX	Nil	[13]
In the aqueous core of liposomes	5 nm Au NPs coated with citrate	DPPC/MPPC/DSPE-PEG-2000	Calcein	514 nm	[15]
	25 nm Au nanorods stabilized with citrate with the aspect ratio around 4.1 (length around 41 nm)	DPPC	Calcein	808 nm	[16]
	Au nanorods (width 25 nm, length 60 nm, CTAB stabilized)	DPPC/Lyso PC/DSPE-PEG or DSPC/DPPC	Calcein	656 nm	[17]
	50–60 nm Au nanostars coated with PEG	DPPC/Lyso PC/DSPE-PEG or DSPC/DPPC	Calcein	850 nm	[17]
	44 nm Au nanostars	DPPC/MPPC/DSPE-PEG2000	Dox	803 nm	[19]
	28–161 nm Au NPs	DSPC	Nil	Nil	[20]
On the surface of liposomes	13 nm Au NPs functionalized with DNA	DOPC/cholesterol/DOPG/MPB-PE	Calcein	302 nm UV	[21]
	4–50 nm Au NPs	DOPC	Nil	Nil	[22]
	Au nanostars	DPPC/MPPC/DSPE-PEG2000	Calcein and PTX	690 nm	[23]
Aggregates with liposomes	20 nm Au NPs	DPPC/DPTAP/cholesterol	Carboxyfluorescein	830 nm	[27]
Free in liposome solution	Au nanorods coated with mPEG-SH 5000	DPPC/DMPC/cholesterol/DSPE-PEG2000	Dox	808 nm	[28]
	PEG coated-multibranched Au nanoantennas	DPPC/MSPC/DSPE-PEG-2000	Dox	808 nm	[29]

### Au NPs within the Lipid Bilayer of Liposomes

**Fig. 2:**
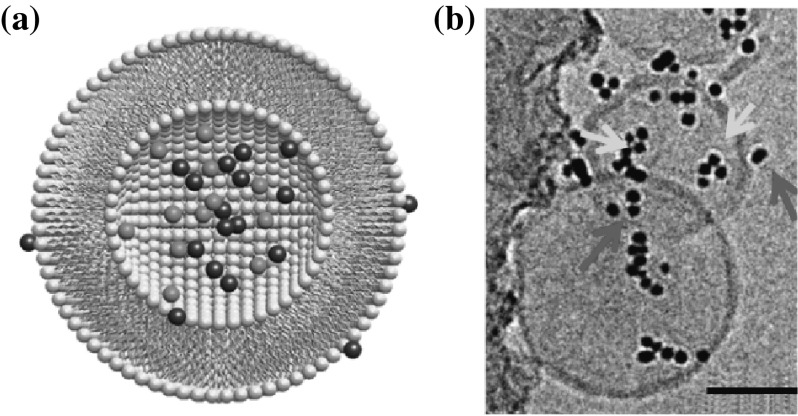
Photosensitive liposomes constructed by encapsulating Au NPs within the aqueous core of thermosensitive liposomes: **a** schematic illustration of the liposomes containing 5 nm Au NPs (gray spheres) and calcein (green spheres); **b** cryo-TEM of liposomes containing Au NPs. Scale bar: 50 nm. *Note*: Au NPs are either encapsulated within liposomes (yellow arrows) or associated with the surface (red arrows). Images are adapted with permission from Ref. [15]. Copyright (2015) Elsevier. (Color figure online)

Park et al. [10] loaded stearylamine-coated Au NPs inside the DPPC bilayers of liposomes using TFH method. In this study, Au NPs had the size of 3–4 nm in diameter (less than the thickness of lipid bilayer, ~5 nm). Loaded liposomes were of relatively narrow-size distribution from 20 to 150 nm through size extrusion. From the magnified ultra-thin film transmission electron microscope (TEM) observations, the liposomes were filled with Au NPs, and more particles were in their edge. As the contents of Au NPs in liposomes were increased, the membrane fluidity was increased (Fig. 2).

To have a higher phase transition temperature than small unilamellar DPPC vesicles, Paasonen et al. [11] synthesized photo-responsive liposomes by associating very small Au NPs (2–3 nm, hexanethiol-capped) with DSPC/DPPC liposomes using REV method that is known to produce large unilamellar vesicles. The lipids and hexanethiol-capped hydrophobic Au NPs were mixed in the organic solvent, to which an aqueous solution containing calcein was added. The solvent was then evaporated to form liposomes encapsulating Au NPs in the lipid bilayer. The diameter of liposomes was between 200 and 500 nm, and the size was not dependent on the lipid composition. The liposomes with *T*_m_ of 45 °C remained intact at 37 °C but a relatively small increase in temperature of the bilayer would increase the permeability of the liposomal membrane, thereby releasing the contents.

Similar structure was also realized using supercritical CO_2_ method with hydrophobic Au NPs (~5 nm). Here the phospholipid lecithin, cholesterin, and hydrophobic Au NPs were mixed in methanol-chloroform solution [12]. The solvent was evaporated to form a thin lipid layer which was later reconstituted with berberine aqueous solution in a high-pressure cell. Supercritical CO_2_ fluid was introduced into the cell followed by incubation at high pressure (16 MPa) and temperature (42.1 °C). After the slow release of CO_2_, the Au NP-liposomes were obtained. Illumination with 250 nm, UV can trigger the release of the encapsulated alkaloid berberine. In another study, paclitaxel (PTX) molecules were first conjugated to Au NPs via PEG ligand containing thiol group, and thus, each Au NP was coated with a layer of PTX molecules making them hydrophobic [13]. The PTX conjugated Au NPs were then encapsulated within the lipid bilayer by TFH technique.

It is important to note that the size of the Au NPs must be in the range of 2–5 nm so that the membrane integrity remains unaffected although it might still cause the considerable distortion of the membrane [14]. These small Au NPs favor renal clearance and are sensitive to UV light, eliciting the controlled drug release from Au NP-liposomes.

### Au NPs in Aqueous Core of Liposomes

Another way to build Au NP-liposome complex is to place hydrophilic Au NPs within the aqueous core of liposomes. The lipids are first dissolved in organic solvent such as chloroform or methanol and form a thin lipid layer by a rotary evaporator under reduced pressure. The lipid film is then reconstituted in an aqueous medium at temperature higher than the phase transition temperature of the phospholipid to form the liposomes. Hydrophilic Au NPs dispersed in the aqueous medium are passively encapsulated within the aqueous core. For example, we encapsulated citrate-coated 5 nm Au NPs within liposomes made of DPPC/MPPC/DSPE-PEG-2000 [15]. Under cryo-TEM, most of Au NPs were either associated with the surface of liposomes or encapsulated within liposomes. By using Nd:YAG pulse laser with a pulse duration of 6 ns and an instantaneous power density approximately equals to 166.67 kW cm^−2^ in each pulse duration, we exposed liposomes with a varying number of pulses in which the pulse frequency was fixed at 1 Hz. Liposomes with Au NPs released the entire drugs within minutes, while less than 40% release was observed in the liposomes without Au NPs under the same conditions.

As the aqueous core of liposomes is in the dimensions of tens to hundreds of nanometers, Au NPs can be bigger than a few nanometers and take a variety of shapes like rod or star. As the Au nanorods (rod-shaped Au NPs) and Au nanostar (star-shaped Au NPs) have strong absorbance in the near-infrared region, this allows the related liposomes to respond to NIR light [16]. Lajunen et al. reported the loading of hydrophilic Au nanorods (25 nm wide; 60 nm long) and Au nanostars (diameter 50–60 nm) in the thermosensitive liposomes composed of DSPC/DPPC and DPPC/LysoPC/DSPE-PEG-2000. These allowed the synthesized liposomes to respond to light (6 W cm^−2^) at wavelengths of 656 and 850 nm for liposomes with Au nanorods and Au nanostars, respectively [17].

The challenging part for this structure is the low efficiency of Au NPs encapsulation in this process. The number of Au NPs in each liposome is also different [18]. A potential solution is to synthesize the particles in situ within liposomes. We demonstrated that Au nanostars containing liposomes could be made by the in situ reduction of gold precursor, HAuCl_4_ (pre-encapsulated within the liposomes) through HEPES diffusion and reduction [19]. The absorption spectra of Au nanostars can be tuned between visible and NIR regions by controlling the size and morphology of Au nanostars through varying the concentrations of HAuCl_4_ and HEPES. These liposomes can produce stronger photoacoustic signals (1.5-fold) in the NIR region than blood. Furthermore, when there were drugs (i.e., doxorubicin (Dox)) within these liposomes, the irradiation with the NIR pulse laser would disrupt the liposomes and trigger the 100% release of these pre-encapsulated drugs within 10 s. Recently, Lee et al. [20] further demonstrated that the reducing agent could be re-encoded before the metal precursors diffused across lipid bilayers self-crystallized to metal nanoparticles in the liposomes.

### Au NPs on the Outer Surface of Liposomes

A third strategy is to locate the Au NPs on the outer surface of the liposomes. The particles are either tethered to the outer surface of the liposomes or directly deposited on the surface of the liposomes surface by lipid vesicle metallization process. On the deposited Au NPs, a gold shell can be further grown.

The tethering of Au NPs to the outer side of the liposomes can be done via DNA hybridization. Dave and Liu synthesized the hybrid nanostructure by complexing two kinds of nanoparticles: 103 nm liposomes conjugated with DNA1 and 13 nm Au NPs functionalized with DNA2 [21]. Under the presence of a linker DNA, Au NPs assembled on the surface of liposomes. The nice thing about this strategy is the easy control of loading density of Au NPs and the distance control between two kinds of nanoparticles that are useful for fundamental understanding on the interaction between light, Au NPs, and liposomes.

In clinical practice, the low-power laser is preferred as it is relatively safe to use and will reduce the damage to the normal tissue/cells. However, a lower-power laser means less heat generated in the process. If the distance between the heat generator (i.e., Au NPs) and lipid bilayer is far, the heat reaching lipid bilayer might be insufficient to destabilize the liposomes. Therefore, it is desired to minimize the distance between Au NPs and lipid bilayer. The solution is to synthesize Au NPs right on the lipid membrane. For example, the 20 µm giant unilamellar vesicles (GUVs) made of DOPC were suspended in a solution of ascorbic acid to which the gold precursor (HAuCl_4_) was added [22]. AA reduced HAuCl_4_ from Au^3+^ to Au^0^ and this zero-valent state led to the deposition of Au NPs on the surface of the liposomes. Similarly, we synthesized Au nanostar-coated liposomes by mixing HEPES buffer and HAuCl_4_ in liposome solution [23], in which the piperazine ring in the HEPES was responsible for the reduction of HAuCl_4_ from Au^3+^ to Au^0^ [24, 25].

These tethered or deposited Au NPs can further act as the seed for the growth of the Au nanoshell on liposomes. For instance, Luo et al. synthesized liposomes composed of soya lecithin and cholesterol and coated liposomes with chitosan. Simultaneously, Au seeds were synthesized using fresh NaBH_4_ and HAuCl_4_ solutions before being mixed with liposomes. HAuCl_4_ and NaBH_4_ were further added into the liposome solution to produce the Au nanoshells on liposomes [26]. The Au shell ensured the stability of the liposomes, preventing any drug leakage at room temperature. However, when there was NIR irradiation, the Au shell converted the photo energy to thermal energy, which induced the instability of liposome membrane and resulted the drug release.

### Assembled as Aggregates with Liposomes

Apart from the above three structures, Au NPs can assemble as aggregates with liposomes. Volodkin et al. [27] made Au NP-liposome assemblies by mixing liposomes and Au NPs. Au NPs were coated with citrate while liposomes were made of DPPC, DPTAP, and cholesterol. Control of the NP aggregation behavior allowed the formation of Au NP-liposome complex structures by means of clustering through single NPs or NP aggregates and created assemblies of types I and II, respectively. Assembly type I, forming at single Au NP and liposomes had diameters near 800 nm. Assemblies of type II were composed from Au NP aggregates of around 300 nm, which had a high cumulative electrostatic charge. The NP aggregates attracted a larger number of liposomes to compensate for the charge excess; this gave bigger assemblies, which were roughly 5 mm in diameter. Single NPs exhibit a surface plasmon resonance in the visible part of the spectrum (at 520 nm) while the aggregates exhibited a red-shifted absorption (650 nm). Under the identical illumination condition, type I assemblies showed no release due to the lower absorption of the non-aggregated nanoparticles. The type II complexes released encapsulated dye upon stimulation with NIR light due to local heating of Au NPs.

### Free in Liposome Solution

Finally, Au NPs can be co-delivered with liposomes and act as heat generators. For example, Dox-containing liposomes constituted of DPPC: DMPC:cholesterol:DSPE-PEG2000 was co-injected with Au nanorods intravenously into a mouse xenograft model of human glioblastoma [28]. Light-mediated drug release was initiated after 48 h allowing sufficient amount for the liposomes and Au nanorods to accumulate at the tumor site. Photothermal conversion was mediated by Au nanorods, increasing the temperature to 43 °C, which in turn triggered the release of Dox. In the same manner, photothermal conversion-mediated Dox release was demonstrated through co-delivery of low-temperature sensitive liposomes (made of DPPC:MSPC:DSPE-PEG-2000) and PEG-coated multibranched Au nanoantennas using NIR irradiation at 808 nm. Such light-mediated drug release localizes the location of drug release thus eliminating the systemic toxicity of free drugs [29].

As Au NPs and drug-containing liposomes are synthesized separately, this type of systems is relatively easier to prepare and more convenient to regulate compared with the above four systems. However, the simple mixing right before administration or the co-administration can not integrate Au NPs and liposomes into one system. Post the administration, Au NPs and liposomes will have different biodistribution and accumulation into tumors. In addition, the stability and integrity of each component during the mixing should be carefully examined as well.

## Mechanisms of Light-Assisted Drug Release from Au NP-Liposomes

Several mechanisms have been proposed as a mean for light-induced membrane destabilization in liposomes to promote cargo release [30]. These include light-induced oxidation, photo-crosslinking, photoisomerization, photocleavage, and photothermal release. However, most Au NP-liposome systems rely on the photothermal effect that is based on the conversion of light into heat to induce liposome permeabilization [31]. Au NPs are able to rapidly and efficiently absorb visible, UV, and NIR light and release energy as heat on the scale of picoseconds. This effect is enhanced especially if the light wavelength matches Au NP absorption bands. This phenomenon generates a heated electron gas that rapidly exchanges energy with the particle structure, which then dissipates this energy in the surrounding medium. When proximal to liposomes, the high temperatures achieved by Au NPs can induce membrane stress and rupture, followed by cargo release. In some cases, instead of membrane rupture, the photothermal effect induces a phase transition in the bilayer, which makes it leaky and leads to an increased cargo release.

So far, both continuous wave and pulsed lasers have given promising results for this kind of controlled release. Rengan et al. [32] synthesized Au-coated DSPC-cholesterol liposomes to perform photothermal cancer treatment. A lethal effect on the breast cancer cells was achieved beyond 4 min of continuous exposure of NIR laser light with 650 mW power. When pulsed laser is used, the cargo release relies on the cumulative action of relatively low energy pulsed illumination, with spectral selectivity controlled by the pulse width. This ensures that photothermal changes affect only the Au NP-liposome complexes, with no shock wave and minimal thermal impact on surrounding medium [33].

While photothermal effect remains the main-stream explanation, the role of photoacoustic conversion is gaining attention. Briefly, when exposed to pulsed laser at the surface plasmon resonance wavelength of Au NPs, there is a rapid thermoelastic expansion of surrounding tissue/solution or microbubble cavitation. This generates pressure impulses that could damage the lipid bilayer of liposomes. Supported by this concept, Wu et al. [34] demonstrated the controlled release from liposomes coated with hollow Au nanoshells with an NIR pulsed laser. We proved the release of both hydrophobic and hydrophilic model drugs (PTX, calcein, and plasmid) through laser excitation at NIR wavelength from liposomes coated with Au NPs and Au nanostars [19, 23, 35]. Recently, Li et al. [36] also uncaged inositol triphosphate (an endogenous cell calcium signaling second messenger) in cell cytoplasm from 2.1 to 5.3 nm Au NP-coated liposomes to activate cell signaling in a nonthermal, ultrafast, and highly controllable fashion.

## Biomedical Applications of Au NP-liposomes

According to the effects of delivered agents, the biomedical applications of Au NP-based photo-responsive liposomes can be grouped as controlled release of molecules and plasmid as a demo, fine-tuning of cellular activities, disease treatment, and theranostics.

### Controlled Release of Molecules and Plasmid as a Demo

Early work in this field mainly focuses on the design and synthesis of the Au NP-based photo-responsive liposomes in which molecules like calcein, fluorescent proteins are used as the model drugs. For example, Volodkin et al. [27] demonstrated the function of Au NP-liposome assemblies by examining the release of fluorescent marker 5(6)-carboxyfluorescein from assembly. Before release, the liposome complex was fluorescent. After 5 s of light exposure, the release took place and the fluorescence intensity of the illuminated part decreased to zero, whereas the fluorescence of the unilluminated area did not change and remained at the same level as that before the exposure. We delivered the plasmid encoding green fluorescence protein (GFP) to the cells with Au nanostar-loaded liposomes (Phospholipon 90G and cholesterol). Without laser treatment, minimal GFP fluorescence can be observed from all other control groups. Following laser activation, however, significant signal enhancement was seen on Au nanostar-loaded liposomes group (1.80-fold) [35].

### Fine-Tuning of Cellular Activities

**Fig. 3:**
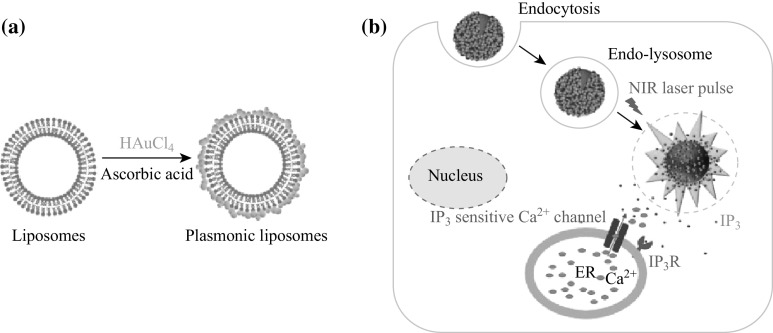
Schematic of the ultrafast near-infrared-light-triggered biomolecule uncaging technique: **a** Formation of plasmonic liposomes; **b** Concept of the near-infrared-light-triggered intracellular uncaging to probe cell signaling. *IP*_*3*_ *R* inositol triphosphate (IP3) receptor; *ER* endoplasmic reticulum. Figures are adapted with permission from Ref. [36]. Copyright (2017) John Wiley and Sons

Model molecules like calcein and GFP greatly help the researchers to optimize and demonstrate the ability of Au NP-liposome complexes in the controlled drug delivery, which also pushes the field to explore other practical applications of these platforms. One direction is to probe cell signaling pathways that occur within milliseconds. Investigation of the signaling kinetics requires faster release of biomolecules with extremely high temporal resolution. Li et al. [36] firstly synthesized NIR-responsive plasmonic liposomes by deposition of Au NPs onto the surface of liposomes via reduction of HAuCl_4_ (Fig. 3a). Au NPs in the range of 2.1–5.3 nm formed discrete Au clusters surrounding the liposome core. They encapsulated inositol triphosphate (IP3) in the liposome complex. IP3 is a second messenger molecule that binds to IP3 receptors (IP3R) located on the endoplasmic reticulum (ER) membrane [37]. The binding event then causes calcium (Ca^2+^) release from the endoplasmic reticulum into the cytosol. For MDA-MB-231 cells labeled with this type of liposomes a single near-infrared laser pulse led to a spontaneous increase in the Fluo-4 fluorescent signal in the laser pulse energy range 38–127 mJ cm^−2^ (Fig. 3b). The fluorescence image sequence showed rapid efflux of Ca^2+^ from internal stores into the cytosol and then propagation into the nucleus. In control groups, no Fluo-4 fluorescence increase was observed for cells treated with noncoated liposomes, suggesting that IP3 was trapped inside liposomes and endolysosomes and was unable to bind to the IP3R in order to trigger the calcium release.

### Cancer Treatment

**Fig. 4:**
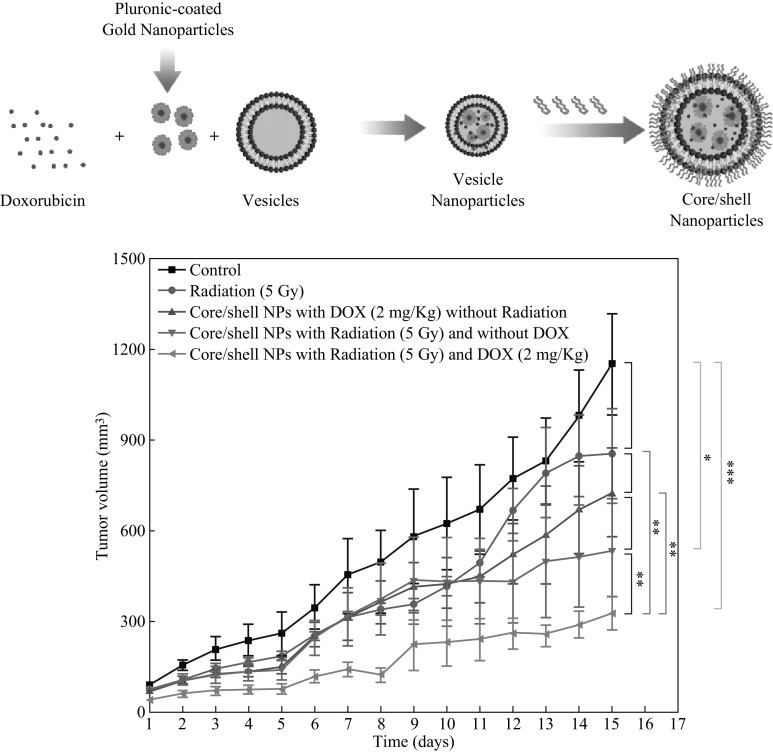
Schematic of the synthesis of core/shell NPs carrying Dox and Au NPs for combination cancer therapy (above) and therapeutic efficacy of various treatments in squamous cell carcinoma cells allograft (bottom). Figures are reprinted with permission from Ref. [39]. Copyright (2016) Elsevier

The greatest interest for the Au NP liposome complex comes from the cancer treatment. Gui et al. [38] synthesized Au nanoclusters and Dox dual-loaded liposomes (AuNCs/Dox-liposome) by using a supercritical CO_2_ method. Both Au and the drugs were embedded in the internal aqueous cavity. They examined the cytotoxicity of this system through the cell experiment with HepG2 cells by comparing the free or loaded Dox, with or without light irradiation. After irradiation for 250 s, a markedly lower cell viability of the AuNCs/Dox-liposome treated cells (4%) was observed compared to the control (60%) and free Dox (10%). This decrease in cell viability should be attributed to the photothermal therapy of the AuNCs/Dox-liposome. Kim et al. [39] encapsulated Au NPs and Dox into liposomes made of l-α-phosphatidylcholine (Fig. 4). In this complex, Au NPs acted as a radiosensitizer to improve the antitumor efficacy of the treatment. In the animal experiment, controls were saline, radiation, Au NP-liposomes with Dox and without radiation, and Au NP-liposomes with radiation and without Dox. A single exposure of radiation-induced apoptosis in a small, defined region of the tumor, resulting in the decrease of tumor size. However, tumor size became similar to control (saline) after 12 days. The mice treated with Au NP-liposomes with radiation without Dox showed more enhanced antitumor efficacy, and the reduced tumor size was maintained throughout the experiment. The most significant antitumor efficacy was observed with combination therapy, which suggests that the core/shell NPs could be used for the combination therapy to treat tumors effectively. Luo et al. [26] prepared Au nanoshells-coated oleanolic acid liposomes mediating by chitosan (GNOLs). The GNOLs presented spherical and uniform size (172.03 nm) with zeta potential (20.7 ± 0.4 mV) and exhibited a slow and controlled release of oleanolic acid at pH 7.4, as well as a rapid release at pH 5.5. In vitro, GNOLs showed significant toxicity to the 143B osteosarcoma cells. Their inhibition rates were 73.74 ± 1.32% at non-irradiation, and 86.91 ± 2.53% at NIR irradiation, respectively. The therapeutic effect of GNOLs on the combined photothermal ablation and chemotherapy was examined with U14 tumor-bearing mice. The tumor size in the group treated with GNOLs plus NIR irradiation showed the highest antitumor efficacy with an inhibition rate of 79.65% during the experiment. The researchers believe GNOLs were a good drug delivery system for effective antitumor therapy. GNOLs accumulated into tumor tissues by the EPR effect; with the NIR irradiation, the gold nanoshells absorbed the light and converted it to hyperthermia, further inducing more OA released rapidly from the GNOLs to realize the most efficient anticancer effect. Meanwhile, the tumor tissues were more sensitive to heat than normal tissues; the tumors were easy to be killed directly by the NIR-induced hyperthermia. This kind of combination treatment of chemophototherapy is emerging as a new treatment option for solid tumors [40].

Finally, Au nanomaterials are well known to be good contrast agents for a variety of optical imaging techniques such as OCT [41], X-ray CT [42], fluorescent imaging [38], photoacoustic imaging [19, 23], and surface plasmon resonance imaging [43]. All Au NP-liposomes can act as the theranostic platforms.

## Challenges and Future

There is an increasing demand for drug delivery system that allows for controlled and triggered release upon stimulation. Such efforts will eventually lead to higher patient survival and better quality of life. The great progress in nanotechnology has paved way for the development of such system. However, for the above systems to be translatable, certain bottlenecks need to be overcome.

The first and foremost concern is the stability of liposomes. Liposomes are well known to be leaky in vivo. It is not well understood whether the incorporation of Au NPs (especially when being embedded in the lipid bilayer and coated on the surface) would decrease the stability of liposomes or make them leakier. To address this concern, in vitro circulation model can be built with microfluidic technologies to examine the stability of the complex in high shear stress.

Second, the photo-responsive liposomal platform is positioned as the drug carriers for superficial tissues such as skin and eyes. Some current systems using Au spherical NPs are developed to react to UV and visible light, which would have limitation in tissue penetration. In addition, the superficial tissues can be damaged if the laser power is too strong. Thus, future research should be focused on the development of Au NP-liposomes that react to low-power NIR.

Finally, the clearance of the delivery system from the human body and long-term effects of chronic usage have to be studied. The incorporation of Au NPs could change the stability, density, and surface chemistry of liposomes, which unavoidably influence the biodistribution and clearance of liposomes. A thorough understanding of their fate in vivo is required.

## Abbreviations

DPPC: Dipalmitoylphosphatidylcholine
DSPC: 1,2-Distearoyl-sn-glycero-3-phosphocholine
MPPC: Myristoylpalmitoylphosphatidylcholine
DSPE-PEG-2000: 1,2-Distearoyl-sn-glycero-3-phosphoethanolamine-*N*-[amino(polyethylene glycol)-2000]
lysoPC: Lysophosphatidylcholines
DOPC: 1,2-Dioleoyl-sn-glycero-3-phosphocholine
DPTAP: 1,2-Dipalmitoyl-3-trimethylammonium propane
DMPC: Dimyristoylphosphatidylcholine
MSPC: Monostearoylphosphatidylcholine
HEPES: 4-(2-Hydroxyethyl)-1-piperazineethanesulfonic acid
